# Local immune response depends on p16^INK4a^ status of primary tumor in vulvar squamous cell carcinoma

**DOI:** 10.18632/oncotarget.17581

**Published:** 2017-05-03

**Authors:** Jacek J. Sznurkowski, Anton Żawrocki, Wojciech Biernat

**Affiliations:** ^1^ Department of Oncological Surgery, The Medical University, Gdańsk, Poland; ^2^ Department of Pathology, The Medical University, Gdańsk, Poland

**Keywords:** vulvar SCC, p16, TILs, TAMs, prognosis

## Abstract

**Background:**

The p16^Ink4a^ is not a surrogate marker for high-risk human papilloma virus (HPV) genotypes but indicates better prognosis in vulvar squamous cell carcinoma patients. Our recent study confirmed substantial mismatch between p16^Ink4a^ and high-risk HPV-status as well as revealed that p16^Ink4a^-overexpression itself is an independent prognostic factor for vulvar cancer.

**Aim:**

To determine significance of the tumor infiltrating immune cells and p16^Ink4a^–status for better outcome of patients with vulvar cancer.

**Methods:**

Intraepithelial tumor infiltrating lymphocytes: CD8+, CD4+, FOXP3+, CD56+, tumor associated macrophages: CD68+, and GZB+ cells were calculated in 85 vulvar squamous cell carcinomas with previously defined p16^Ink4a^ and high-risk HPV-status. Number of intraepithelial CD8+, CD4+, FOXP3+, CD56+, CD68+ and GZB+ cells were compared between tumors with different p16^INK4a^ status and overlapping high-risk HPV-status separately. Survival analyses included the Kaplan–Meier method, log-rank test and Cox proportional hazards model.

**Results:**

p16^Ink4a^-negative tumors were more infiltrated by intraepithelial CD8+, CD4+ and GZB+ cells than p16^Ink4a^-positive tumors (p=0.032, p=0.016 and p=0.007 respectively). High-risk HPV-status did not correlate with the infiltration of immune cells. Median follow up was 89.20 months (range 1.7-189.5). High CD4+ and CD56+ indices were correlated with prognosis in p16^Ink4a^–positive cases (p=0.039 and p=0.013 respectively). Low CD68+ infiltrates were correlated with prognosis in p16^Ink4a^-negative cases (p=0.018). Conclusion: p16^Ink4a^-status impacts local immune surveillance as represented by tumor infiltrating immune cells. Immunologic effects contributing to clinical outcome might depend on p16^Ink4a^-overexpression.

## INTRODUCTION

Vulvar cancer represents 3-5% of all gynecological malignancies with an incidence rate 1-2 per 100,000 women per year. The most common type is vulvar squamous cell carcinoma (vSCC) [1]. There are two distinct aetiopathogenic pathways involved in the induction of vSCC. The first one involves the presence of transforming infections with high-risk (hr) HPV- genotypes, the latter arises in the absence of HPV during chronic dermatosis [2]. Intracellular release of viral oncoproteins E6 and E7 disturbs regulation of the cell cycle and coexists with nuclear and cellular accumulation of cyclin-dependent kinase inhibitor p16^Ink4a^ [2, 3].

Although demonstration of overexpression of p16^Ink4a^ serves as a surrogate marker for a transforming infection with HPV-high-risk genotypes in cervical cancer [4], the largest up to date cohort study revealed that p16^Ink4a^ is not a substitute marker for (hr) HPV-DNA in vSCC [5].

Meta-analysis of 2309 vSCC cases indicated that p16^Ink4a^ status correlates with better prognosis of vulvar cancer patients [6]. It was also shown that patients with cancers presenting p16^Ink4a^-overexpression are very sensitive to radiotherapy and this could explain their better outcome [7].

Indeed, our recent study on 85 vSCCs confirmed substantial mismatch between p16^ink4a^ and HPV-status and revealed independent prognostic significance exclusively for p16^Ink4a^ -overexpression. This study has also revealed that the overexpression of p16^Ink4a^ predicts improved prognosis for patients who have undergone adjuvant radiotherapy [8].

The impact of p16^Ink4a^ -status on vulvar cancer immune surveillance as represented by tumor-infiltrating lymphocytes (TILs) and tumor associated macrophages (TAMs) is unknown and potentially could be correlated with the outcome of patients.

TILs are considered to be a manifestation of the host immune response against cancer cells [9] and consist of two major populations of effector cells: the CD8+ T lymphocytes and the natural killer (NK) cells [10]. Another subset of TILs is composed of CD4+ T lymphocytes which play a central role in initiating, maintaining and modulating anticancer immune responses [11, 12]. Functional analysis of CD4+ T cells has recognized CD25+FOXP3+CD4+ Tregs cells which have been found to suppress the tumor specific immune response [13, 14].

Innate anti-tumor immunity is primarily performed by natural killer (NK) (CD3−CD56+) and NK/T (CD3+CD56+) as well as soluble factors abundant in the tumor microenvironment [15].

Another component of local immunologic response exerting inhibitory functions of other immune cells are TAMs (CD68+ cells). Their function depends on the release of inhibitory cytokines or reactive oxygen species (ROS) [16].

Crucial for cytotoxic function of all immune cells are granzymes, a family of serine proteases. Granzyme B (GZB) is one of the most commonly present in the cytotoxic lymphocytes and the GZB-induced cell death has been regarded as a primary mechanism utilized by adaptive (CD8+) as well as innate (NK/NKT) effectors to eliminate cancer cells [17, 18].

To look for the immunological explanation of better prognosis of p16^INK4a^-positive patients we aimed to compare local immune surveillance (represented by TILs: CD8+, CD4+, FOXP3+, CD56+; TAMs: CD68+, and GZB+ cells) in cases having tumors with various p16^INK4a^ -status as well as to analyze the correlation of these immunomodulating factors with the survival in both p16^Ink4a^ -negative and p16^Ink4a^ -positive groups of vSCC patients.

## RESULTS

### Characteristics of patients with opposed p16^INK4a^ and (hr) HPV-DNA statuses

Comparison of clinicopathological features of patients having primary tumors: negative and positive for p16^INK4a^ and (HR) HPV-DNA revealed lack of differences between groups.

P16^INK4a^ and (hr) HPV-DNA status of vulvar cancer did not correlate with age at diagnosis, depth of invasion, tumor grade, pT, pN, FIGO stage and recurrence (Table 1).

**Table 1:** Comparison of Clinicopathological features between patients having primary tumors: negative and positive for p16 and (HR) HPV-DNA

Clinicopathological feature	p16 staining- status		p	(HR) HPV status		p
	Negative (n=50)	Positive (n=35)		Negative (n=48)	Positive (n=37)	
Age,/median/	70	65	0.306	68	67	0.669
Depth of invasion,/median/	7.03	7.5	0.228	7.0	7.9	0.096
G1/G2+G3	13/37	15/20	0.159	15/33	13/24	0.817
G1/G2/G3	13/26/11	15/11/9	0.142	15/23/10	13/14/10	0.630
pT (1/2/3)	46/4/0	30/4/1	0.411	46/2/0	30/6/1	0.081
Meta+/meta-	24/26	15/20	0.665	20/28	19/18	0.390
FIGO stage I/II/III/IV	26/0/21/3	18/2/14/1	0.346	28/0/19/1	16/2/16/3	0.158
Recurrence +/−	11/39	5/30	0.413	7/41	9/28	0.277

### Immunohistochemistry for immune cells

**Figure 1:**
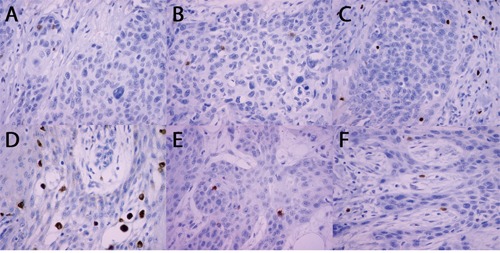
Microphotographs of immunohistochemical staining for subtypes of immune cells **(A)** CD4+; **(B)** CD8+; **(C)** FOXP3+; **(D)** CD68+; **(E)** CD56+; **(F)** GZB+.

CD4+, CD8+, FOXP3+, CD68+, CD56+ and GZB+ cells were detected within cancer cell nests or the mesenchymal stroma. Figure 1A-1F shows micro-photographs of immunohistochemical staining for all subtypes of immune cells within cancer nests.

P16^INK4a^-positive primary tumors had cancer nests less infiltrated with adaptive immune effectors: CD8+ (p=0.032), CD4+ (p=0.016) T lymphocytes as well as GZB+ cells (indicating combined cytolytic power of innate and adaptive TILs) (p=0.007).

(hr) HPV-status did not impact the infiltration of TILs. Detailed data on the number of particular subtypes of immune cells within cancer nests in relation to p16^INK4a^ and (hr) HPV status of the primary tumor includes Table 2.

**Table 2:** Number of TILs within cancer nest in general cohort and in relation to p16 as well as (hr) HPV-DNA status of the primary tumor

TILs	General cohort (n=85)	p16 positive (n=35)	p16 negative (n=50)	pUMW	(hr) HPV-DNA positive (n=37)	(hr) HPV-DNA negative (n=48)	pUMW
CD4+ median (range)	2.67 (0-21.67)	0 (0-18.33)	4.16 (0.0-21.67)	**0.016**	2.67 (0-19.67)	2.67 (0-21.67)	0.594
CD8+ median (range)	16.67 (0-214.33)	12 (0-121)	21.17 (0.0-214.33)	**0.032**	12.33 (0-121)	20.33 (0-214.33)	0.158
CD56+ median (range)	2 (0-37.0)	1.5 (0-37)	2 (0-13.67)	0.202	1.83 (0-37)	2 (0-13.67)	0.783
CD68+ median (range)	8 (0-20.33)	7 (0-19)	8.67 (0-20.33)	0.140	7.67 (0-10.67)	8.33 (0-20.33)	0.979
FOXP3+ median (range)	15 (0-66.0)	11.4 (0-58.4)	16.5 (0-66.0)	0.158	11 (0.75-58.4)	17 (0-66)	0.131
GzB+ median (range)	3 (0-14.33)	1.67 (0-14.33)	3.33(0-13.33)	**0.007**	3 (0-13.33)	3 (0-14.33)	0.669

### Prognostic significance of TILs in relation to p16^INK4a^ -status of the primary tumor

Patients were divided into groups CD8+, CD4+, FOXP3+, CD68+, CD56+, GZB+ positive and CD8+, CD4+, FOXP3+, CD68+, CD56+, GZB+ negative based on median value of infiltrating subtypes of TILs (cut off point) in entire cohort of vSCC cases (Table 2).

**Figure 2:**
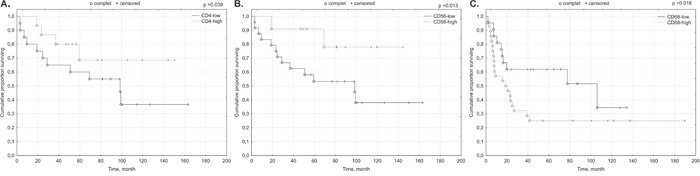
Prognostic significance of TILs in relation to p16 status of the primary tumor **(A)** CD4+; **(B)** CD56+; **(C)** CD68+.

High density of CD4+ and CD56+ lymphocyte infiltrates within p16^INK4a^-positive cancer nests tumors correlated with better outcome in patients (p=0.039, p=0.013) (Figure 2A-2B).

In p16-negative tumors inverse correlation of high (IE) CD68+ infiltrates with prognosis was notified (p=0.018) (Figure 2C).

### Correlation between subtypes of TILs and clinicopathological features of p16-positive and negative cases

CD56+ and CD68+ infiltrates were correlated with depth of invasion in p16-positive (r= 0.359, p=0.037 and r=0.608, p=0.001, respectively) and p16-negative tumors (r=0.392, p=0.005 and r=0.360, p=0.011 respectively). Depth of invasion was also correlated with infiltration of GZB+ cells but this was only observed within p16-positive tumors (r=0.427, p=0.012).

In p16-negative tumors, CD68+ infiltrates were correlated with metastases and higher stage of disease (r=0.464, p=0.001 and r=0.475, p=0.001, respectively). CD56+ cells were correlated with lower differentiation grade within p16-positive tumors (r=0.475, p=0.012).

Detailed correlations of all evaluated immune cells with common clinicopathological parameters are contained in the Supplementary Table 1 (p16-negative cases) and Supplementary Table 2 (p16-positive cases).

## DISCUSSION

To look for the explanation of the prognostic significance of p16^Ink4a^-overexpression within vulvar cancer tissue [6, 8] we compared at first common clinicopathological features between patients with opposed p16^Ink4a^ status. Keeping with the findings of other studies [24, 25], our results confirmed that p16^Ink4a^ -overexpression is not correlated with any clinicopathological feature potentially influencing survival of vulvar cancer patients (Table 1).

Further, we decided to assess the relation of p16^Ink4a^ status of the primary tumor with immunomodulating factors.

To compare cancer immune surveillance (represented by TILs: CD8+, CD4+, FOXP3+, CD56+, TAMs: CD68+ and GZB+ cells) in p16^Ink4a^ -positive and negative primary tumors we analyzed mononuclear infiltrates within cancer nests only.

We found that p16^Ink4a^-negative cancer nests were more infiltrated by effectors of adaptive immune response (CD8+, CD4+ T lymphocytes) and demonstrated higher cytotoxic activity of immune cells (GZB+) while p16^Ink4a^ status has no impact on number of innate effectors (CD56+) and regulatory (FOXP3+, CD68+) infiltrates. Interestingly, (hr) HPV-status did not impact the infiltration of subtypes of TILs (Table 2).

Lower TILs indices may reflect lower local immunogenicity of p16^Ink4a^-positive tumors. The varying extent of enrichment of p16^Ink4a^-positive and -negative tumors in the number of CD8+ and CD4+ cells may be a consequence of diverse expression of tumor associated antigens (TAAs), MHC antigens, costimulatory molecules, and the statuses of antigen-presenting cells (APCs) [16].

Further investigation is necessary for elucidation of tumor microenvironment biological characteristics responsible for infiltration.

Higher cytotoxic activity of the immune system (GZB+ infiltrates) was found in p16^Ink4a^-negative cancer nests. These nests were more intensely infiltrated only by CD8+ cells, therefore, granzyme B-dependent elimination of tumor cells seems to be major action of CD8+ cells rather than CD56+ in vulvar cancer surveillance. Interestingly, with exception of CD68+ cells, all other subtypes of TILs infiltrating p16^Ink4a^-negative cancers did not influence the clinical outcome of patients. High density of CD68+ cell infiltrates was correlated with disseminated disease and predicted shorter survival in this group. The current literature supports the hypothesis that TAMs play an active role in both tumor immunosuppression and promotion of tumor growth [16].

Within p16^Ink4a^-positive tumors which were less infiltrated with adaptive immune effectors (CD4+, CD8+ lymphocytes), two subtypes of immune cells (CD4+ and CD56+) were found to be correlated with longer overall survival (OS).

CD4+ T cells may enhance anticancer effect by providing help to CD8+ T cells through facilitation of dendritic cell (DC) - T cell interactions [16] or, directly recognizing endogenously processed antigens displayed on the surface of tumors followed by secretion of type 1 cytokines [26, 27] or direct cancer cell killing [28]. Which of these immunologic effects is contributing to improved clinical outcomes in vSCC requires further investigation.

Interestingly facilitation of dendritic cell (DC) - T cell interactions, was also suggested to be primary biological role of CD56+ cells rather than direct elimination of tumor targets [29].

Recent studies suggest that, besides the granzyme-mediated lysis, the CD56+ cells may induce apoptosis in a broad variety of tumor cells by means of expression of several TNF-family ligands [30].

However, most of the data were retrieved from our previous studies, originality of current report is supported by completely new study project and design. The impact of p16^Ink4a^-status on tumor immune infiltrates has never been analyzed before. This study provides a new insight into immune surveillance on vSCC as for the first time demonstrates that p16^Ink4a^-overexpression impacts the number of TILs and TAMs at primary site.

Lack of prognostic significance of adaptive immune effectors and regulatory T cells described by others [31] was previously confirmed by our group for nearly the same cohort [19, 20]. All these findings have suggested no place for immunotherapy for vulvar cancer patients.

In the light of current results such a conclusion should be reconsidered as we have proven that from the immunologic point of view, vSCC seems to consist of two separate entities dependent on p16^Ink4a^ status with different subtypes of prognostic immune infiltrates.

The weaknesses of the current study are the retrospective design and the small size of cohort involved. Its strength lies in the consistency of the treatment of patients under uniform standards and their long observation, revealing recurrences and hence enabling the assessment of the prognostic significance of all the analyzed biomarkers.

## PATIENTS AND METHODS

This retrospective study was approved by the Polish Ministry of Science and Higher Education review board, who determined that further informed consent was not required as informed consent for tissue sampling was obtained from all patients prior to surgical treatment and written consent was given by the patients for their information to be stored in the hospital database and used for research.

### Population

Recently described by our group, a cohort of 85 patients with known p16^INK4a^ and (hr) HPV status, was included into study analyses [8]. Briefly, the median age of the patients was 68 years (range 36-85), the median duration of follow-up was 89,20 months (range 1.7-189.5), The 5-year disease free survival (DFS) rate was 61.75 % [8].

### Tissue samples

We analyzed all 85 primary tumors for TAMs represented by CD68-positive cells and 9 for TILs as represented by: CD8+, CD4+, FOXP3+, CD56+ and GZB+ cells. Data on TILs in remaining 76 vSCCs were retrieved from our previous studies [19, 20, 21]. Data on p16^INK4a^ and (hr) HPV status were retrieved from our recent study [8].

### Antibodies

Mouse anti-human monoclonal antibodies against CD4 (NCL-L-368), CD8 (NCL-L-295), CD56 (NCL-CD56-1B), were obtained from Novocastra, Inc. Mouse anti-human monoclonal antibody against FOXP3 (cat. No ab20034) and CD68 (cat. No ab955) were obtained from Abcam, Inc. Mouse anti-human polyclonal antibody against Granzyme B (cat. No 760-4283) was obtained from Ventana Medical Systems, Inc. Mouse anti-human monoclonal antibody against p16 (cat. No sc-56330) was obtained from Santa Cruz Biotechnology Inc., USA. Detailed antibodies characteristics including dilution are contained in the Supplementary Table 3.

### Immunohistochemistry

The immunohistochemical staining for all antibodies: p16, CD8, CD4, FOXP3, CD68, CD56, and GZB was performed according to the following protocol. Four μm-thick serial sections were cut, deparaffinized and subjected to a heat-induced epitope retrieval step before being incubated with the primary antibodies. Sections were immersed in Target Retrieval Solution (pH 6,0; Dako Cytomation, Denmark) and heated in a pressure cooker. The slides were incubated for 90 minutes with the primary antibodies.

The reaction was visualized by the Novolink polymer detection system (Novocastra Laboratories). Appropriate positive (normal tonsil for CD4, CD8, FOXP3, granzyme B, CD56, CD68, HPV-related cervical cancer for p16) and negative controls (the primary antibody was replaced with normal mouse IgG at an appropriate dilution) were included for each case. The results of immunohistochemistry were evaluated by two independent pathologists who did not have any knowledge of the clinical data. The concordance rate between their observations was over 96%.

### Evaluation and classification of TILs: CD8+, CD4+, FOXP3+, CD68+, CD56+ and GZB+ cells

The degree of immune cell infiltration was determined in more than 10 high-power (x400) microscopic fields for each tissue sample. Then, five areas with the most dense lymphocyte distribution (hot spots) were selected and micro-photographs were taken. The quantitative analysis was performed with Multiscan 14.2 software. The number of *CD8+, CD4+, FOXP3+, CD68+, CD56+ and GZB+ cells* was counted exclusively within primary tumor cancer cell nest. For each case the mean index of particular subtype of intraepithelial (IE) TILs per single high power field was counted and then statistically analyzed.

Patients were divided into *CD8+, CD4+, FOXP3+, CD68+, CD56+, GZB+ cells* low and high intensity group (lowCD8+, lowCD4+, lowFOXP3+, lowCD68+, lowCD56+, lowGZB+ and highCD8+, highCD4+, highFOXP3+, highCD68+, highCD56+, highGZB+ respectively) based on median cell number (cut-off point) to analyze the prognostic significance of particular subtypes of (IE) TILs [19–23].

### Evaluation and classification of p16^ink4a^ immunostaining

The evaluation of the p16^ink4a^ immunostaining was performed on 3 different staining patterns: negative, focal, and diffuse staining. For statistical purposes, p16 immunostaining was classified as positive or negative. Staining for p16^ink4a^ was considered positive only in cases with a strong diffuse and continuous nuclear/cytoplasmic expression of p16^ink4a^ within the cancer nests (focal and weak diffuse staining were considered negative) [8].

### Detection of DNA HPV

Tissue dissection and DNA preparation as well as mucosal HPV DNA amplification and genotyping were detailed for this cohort in our recent study [8].

### Statistical analysis

The statistical analysis was performed using chi-square test or Fisher's exact probability test, Kruskal-Wallis test. Correlations and differences between variables were assessed using the Spearman's rank correlation coefficient.

Overall survival curves were estimated by the Kaplan-Meier method and compared by the two-sided log-rank test. For uni- and multivariate analyses the Cox proportional-hazards regression model was used to explore the impact of individual variables on survival.

P-values of <0.05 were regarded as significant in all of the analysis. All analyses were performed with the statistical software Statistica 10 (Stat Soft Inc.).

## CONCLUSION

Different immune cells infiltration and diverse prognostic significance of subtypes of TILs discovered in patients with p16^INK4a^ -positive and negative cancers suggest that local immune surveillance and immunologic effects contributing to clinical outcome depend on cancer p16^INK4a^ status. Our results suggest that not HPV infection itself but p16^INK4a^ overexpression contributes to shaping of the tumor microenvironment and p16^INK4a^ -status should stratify patients for separate immunotherapeutic approaches in vSCC.

## SUPPLEMENTARY TABLES



## References

[1] Beller U, Quinn MA, Benedet JL, Creasman WT, Ngan HY, Maisonneuve P, Pecorelli S, Odicino F, Heintz AP (2006). Carcinoma of the vulva. In J Gynaecol Obstet.

[2] Del Pino M, Rodriguez-Carunchio L, Ordi J (2013). Pathways of vulvar intraepithelial neoplasia and squamous cell carcinoma. Histopathology.

[3] Santos M, Landolfi S, Olivella A, Lloveras B, Klaustermeier J, Suárez H, Alòs L, Puig-Tintoré LM, Campo E, Ordi J (2006). P16 overexpression identifies HPV-positive vulvar squamous cell carcinomas. Am J Surg Pathol.

[4] Bergeron C, Ronco G, Reuschenbach M, Wentzensen N, Arbyn M, Stoler M, von Knebel Doeberitz M (2015). The clinical impact of using p16(INK4a) immunochemistry in cervical histopathology and cytology: an update of recent developments. Int J Cancer.

[5] de Sanjosé S, Alemany L, Ordi J, Tous S, Alejo M, Bigby SM, Joura EA, Maldonado P, Laco J, Bravo IG, Vidal A, Guimerà N, Cross P (2013). Worldwide human papillomavirus genotype attribution in over 2000 cases of intraepithelial and invasive lesions of the vulva. Eur J Cancer.

[6] Cao H, Wang S, Zhang Z, Lou J (2016). Prognostic value of overexpressed p16INK4a in vulvar cancer: a meta-analysis. PLoS One.

[7] Lassen P, Eriksen JG, Hamilton-Dutoit S, Tramm T, Alsner J, Overgaard J (2009). Effect of HPV-associated p16INK4A expression on response to radiotherapy and survival in squamous cell carcinoma of the head and neck. J Clin Oncol.

[8] Sznurkowski JJ, Zawrocki A, Biernat W (2016). The overexpression of p16 is not a surrogate marker for high-risk human papilloma virus genotypes and predicts clinical outcomes for vulvar cancer. BMC Cancer.

[9] Rosenberg SA (2001). Prognosis in human tumor immunology and immunotherapy. Nature.

[10] Abbas AK, Lichtman AH, Pober JS (2003). Immunity to tumors. Cellular and Molecular Immunology.

[11] Toes RE, Ossendorp F, Offringa R, Melief CJ (1999). CD4 T cells and their role in antitumor immune responses. J Exp Med.

[12] Cohen PA, Peng L, Plautz GE, Kim JA, Weng DE, Shu S (2000). CD4+ T cells in adoptive immunotherapy and the indirect mechanism of tumor rejection. Crit Rev Immunol.

[13] Sakaguchi S, Sakaguchi N, Shimizu J, Yamazaki S, Sakihama T, Itoh M, Kuniyasu Y, Nomura T, Toda M, Takahashi T (2001). Immunologic tolerance maintained by CD25+CD4+ regulatory T cells their common role in controlling autoimmunity, tumor immunity, and transplantation tolerance. Immunol Rev.

[14] Hori S, Noura T, Sakaguchi S (2003). Control of regulatory T cell development by the transcription factor Foxp3. Science.

[15] Whiteside TL, Vojanovic NL, Herberman RB (1998). Natural killer cells and tumor therapy. Curr Top Microbiol Immunol.

[16] Whiteside TL (2010). Immune responses to malignancies. J Allergy Clin Immunol.

[17] Rousalova I, Krepela E (2010). Granzyme B-induced apoptosis in cancer cells and its regulation (review). Int J Oncol.

[18] Grossman WJ, Verbsky JW, Tollefsen BL, Kemper C, Atkinson JP, Ley TJ (2004). Differential expression of granzymes A and B in human cytotoxic lymphocyte subsets and T regulatory cells. Blood.

[19] Sznurkowski JJ, Zawrocki A, Emerich J, Biernat W (2011). Prognostic significance of CD4+ and CD8+ T cells infiltration within cancer cell nests in vulvar squamous cell carcinoma. Int J Gynecol Cancer.

[20] Sznurkowski JJ, Zawrocki A, Emerich J, Sznurkowska K, Biernat W (2011). Expression of indoleamine 2,3-dioxygenase predicts shorter survival in patients with vulvar squamous cell carcinoma (vSCC) not influencing on the recruitment of FOXP3-expressing regulatory T cells in cancer nests. Gynecol Oncol.

[21] Sznurkowski JJ, Zawrocki A, Biernat W (2014). Subtypes of cytotoxic lymphocytes and natural killer cells infiltrating cancer nests correlate with prognosis in patients with vulvar squamous cell carcinoma. Cancer Immunol Immunother.

[22] Wakabayashi O, Yamazaki K, Oizumi S, Hommura F, Kinoshita I, Ogura S, Dosaka-Akita H, Nishimura M (2003). CD4+ T cells in cancer stroma, not CD8+ T cells in cancer cell nests, are associated with favorable prognosis in human non-small cell cancers. Cancer Sci.

[23] Mansfield AS, Heikkila PS, Vaara AT, von Smitten KA, Vakkila JM, Leidenius MH (2009). Simultaneous Foxp3 and IDO expression is associated with sentinel lymph node metastases in breast cancer. BMC Cancer.

[24] Alonso I, Fusté V, del Pino M, Castillo P, Torné A, Fusté P, Rios J, Pahisa J, Balasch J, Ordi J (2011). Does human papillomavirus infection imply a different prognosis in vulvar squamous cell carcinoma?. Gynecol Oncol.

[25] Tringler B, Grimm C, Dudek G, Zeillinger R, Tempfer C, Speiser P, Joura E, Reinthaller A, Hefler LA (2007). P16INK4a expression in invasive vulvar squamous cell carcinoma. Appl Immunohistochem Mol Morphol.

[26] Friedman KM, Prieto PA, Devillier LE, Gross CA, Yang JC, Wunderlich JR, Rosenberg SA, Dudley ME (2012). Tumor specific CD4+ melanoma tumor-infiltrating lymphocytes. J Immunother.

[27] Quezada SA, Simpson TR, Peggs KS, Merghoub T, Vider J, Fan X, Blasberg R, Yagita H, Muranski P, Antony PA, Restifo NP, Allison JP (2010). Tumor-reactive CD4(+) T cells develop cytotoxic activity and eradicate large established melanoma after transfer into lymphopenic hosts. J Exp Med.

[28] Kelly JM, Darcy PK, Markby JL, Godfrey DI, Takeda K, Yagitab H, Smyth MJ (2002). Induction of tumor-specific T cell memory by NK cell-mediated tumor rejection. Nat Immunol.

[29] Vujanovic NL, Nagashima S, Herberman RB, Whiteside TL (1996). Non-secretory apoptotic killing by human natural killer cells. J Immunol.

[30] Donia M, Hansen M, Sendrup SL, Iversen TZ, Ellebæk E, Andersen MH, Straten PT, Svane IM (2013). Methods to improve adoptive T-cell therapy for melanoma: IFN-gamma enhances anticancer responses of cell products for infusion. J Invest Dermatol.

[31] de Jong RA, Toppen NL, Ten Hoor KA, Boezen HM, Kema IP, Hollema H, Nijman HW (2012). Status of cellular immunity lacks prognostic significance in vulvar squamous carcinoma. Gynecol Oncol.

