# Supplementary material for: Local immune response depends on p16INK4a status of primary tumor in vulvar squamous cell carcinoma
Source: Oncotarget. 2017 May 3;8(28):46204–10. doi: 10.18632/oncotarget.17581 (PMC5542260; doi:10.18632/oncotarget.17581)
Supplement: Supplementary file 1 [file oncotarget-08-46204-s001.pdf]

Local immune response depends on p16^{INK4a} status of primary tumor in vulvar squamous cell carcinoma

SUPPLEMENTARY MATERIALS

Supplementary Table 1: Correlation between subtypes of tumor infiltrating immune cells and clinicopathological features of p16-negative vSCC cases

Clinicopathological feature	P-16-negative tumor (n=50)											
	CD4+	p	CD8+	p	FOXP3+	p	CD56+	p	GZB+	p	CD68+	p
Depth of invasion./ median/	-0.171	0.240	0.026	0.858	-0.014	0.925	0.392	**0.005**	0.146	0.317	0.360	**0.011**
G1/G2+G3	-0.156	0.278	-0.066	0.647	-0.160	0.268	0.234	0.102	0.092	0.526	0.129	0.376
G1/G2/G3	-0.233	0.104	-0.012	0.934	-0.072	0.621	0.207	0.149	0.038	0.792	0.151	0.299
pT (1/2/3)	-0.112	0.438	-0.084	0.560	-0.092	0.525	0.031	0.832	0.085	0.560	0.003	0.986
Meta+/meta-	-0.214	0.136	-0.104	0.472	0.049	0.738	0.246	0.085	0.160	0.267	0.464	**0.001**
FIGO stage I/II/ III/IV	-0.232	0.105	-0.129	0.370	0.011	0.937	0.233	0.104	0.215	0.133	0.475	**0.001**

Supplementary Table 2: Correlation between subtypes of tumor infiltrating immune cells and clinicopathological features of p16-positive vSCC cases

Clinicopathological feature	P-16 positive tumors (n=35)											
	CD4+	p	CD8+	p	FOXP3+	p	CD56+	p	GZB+	p	CD68+	p
Depth of invasion./ median/	-0.030	0.863	-0.213	0.219	-0.187	0.283	0.359	**0.037**	0.399	**0.019**	0.608	**0.001**
G1/G2+G3	0.071	0.686	-0.080	0.647	-0.080	0.648	-0.086	0.629	0.427	**0.012**	0.246	0.154
G1/G2/G3	0.018	0.920	-0.109	0.535	-0.168	0.334	-0.154	0.384	0.284	0.103	0.156	0.372
pT (1/2/3)	0.023	0.895	0.285	0.097	0.045	0.799	0.117	0.509	0.014	0.939	0.067	0.702
Meta+/meta-	0.012	0.944	-0.263	0.127	-0.252	0.145	0.058	0.744	0.098	0.583	0.160	0.358
FIGO stage I/II/ III/IV	-0.008	0.965	-0.143	0.411	-0.284	0.098	0.052	0.769	0.076	0.669	0.154	0.377

Supplementary Table 3: Detailed antibodies characteristic

Antibody		Clone (nr Cat.)	Supplier	Dilution
CD4	4B12	Cat. No NCL-L-CD4-368	Novocastra	1:80
CD8	1A5	Cat. No NCL-L-CD8-295	Novocastra	1:80
FOXP3	263A/E7	Cat. No ab20034	Abcam	1:800
CD56	1B6	Cat. No NCL-CD56-1B6	Novocastra	1:400
Granzyme B	Polyclonal	Cat. No 760-4283	Ventana	RTU
CD68	KP1	Cat. No ab955	Abcam	1:400
P16	JC8	Cat. No sc-56330	Santa Cruz	1:200
